# Increased level and fragmentation of plasma circulating cell-free DNA are diagnostic and prognostic markers for renal cell carcinoma

**DOI:** 10.18632/oncotarget.24943

**Published:** 2018-04-17

**Authors:** Yoshiyuki Yamamoto, Motohide Uemura, Kosuke Nakano, Yujiro Hayashi, Cong Wang, Yu Ishizuya, Toshiro Kinouchi, Takuji Hayashi, Kyosuke Matsuzaki, Kentaro Jingushi, Taigo Kato, Atsunari Kawashima, Takeshi Ujike, Akira Nagahara, Kazutoshi Fujita, Ryoichi Imamura, Norio Nonomura

**Affiliations:** ^1^ Department of Urology, Osaka University Graduate School of Medicine, Suita 565-0871, Japan; ^2^ Department of Therapeutic Urologic Oncology, Osaka University Graduate School of Medicine, Suita 565-0871, Japan

**Keywords:** circulating cell-free DNA, renal cell carcinoma, plasma, level, fragment size

## Abstract

**Background:**

Reliable biomarkers for renal cell carcinoma (RCC) have yet to be found. Circulating cell-free DNA (cfDNA) is an emerging resource for the diagnosis and prognosis of various cancers. This study aims to identify novel blood biomarkers for RCC.

**Materials And Methods:**

Plasma cfDNA was extracted from RCC patients (*n* = 92) and healthy controls (*n* = 41). Levels of cfDNA were determined using quantitative real-time PCR of ACTB as the target gene, and cfDNA fragment size was measured using a microfluidics-based platform. Diagnostic potential was assessed using receiver operating characteristic (ROC) and logistic regression analysis, and prognostic potential was evaluated using log-rank test.

**Results:**

Median levels of cfDNA from RCC patients were significantly higher than those from healthy controls (3803 vs 2242 copies/ml, *p* < 0.001). Median fragment sizes of cfDNA in RCC patients were shorter than those in healthy controls (170 vs 171 bp, *p* = 0.052). To evaluate level of cfDNA as a diagnostic tool for RCC, ROC curve analysis revealed a sensitivity of 63.0% and a specificity of 78.1%. Multivariate analysis indicated that age, gender and the level of cfDNA were significantly associated with the presence of RCC (*p* < 0.001, *p* = 0.013, *p* < 0.001, respectively). Additionally, shorter cfDNA fragment size was negatively associated with progression-free survival (*p* = 0.006).

**Conclusions:**

Our study demonstrates the diagnostic and prognostic potential of plasma cfDNA as a biomarker for RCC.

## INTRODUCTION

Renal cell carcinoma (RCC) is the seventh most common cancer and comprises 2.4% of all adult malignancies worldwide [1]. The 5-year specific survival is reported to be 71%, although 30% of RCC patients present with the evidence of distant metastasis at initial diagnosis, which is associated with the poor prognosis for patients in an advanced stage [2, 3]. Currently, radiological examinations are commonly applied for the diagnosis of RCC and are subsequently confirmed by histopathological examinations. However, these examinations can be problematic; radiological examinations are insufficient for qualitative diagnosis of tumor, and histopathological examinations are invasive, unrepeatable, and are not suited for disease monitoring.

Blood-based tests, also known as liquid biopsy, can offer a potential alternative measure that overcomes the problems posed by traditional methods. Liquid biopsy such as circulating tumor cells or circulating cell-free DNA (cfDNA) constitutes a promising and less invasive technique [4]. However, no satisfactory blood-based markers for RCC currently exist, creating an urgent need for the identification of new molecular markers. CfDNA is released from both normal and tumor cells by different molecular processes, such as cell apoptosis, necrosis and secretion of genomic DNA fragments [5]. Generally, cfDNA fragment size falls within a range of multiples of 180 bp, consistent with the unit size of nucleosomes, similar to DNA from apoptotic cells [5, 6]. The abundance and relative fragmentation of cfDNA in blood can be a universal marker for RCC [7–13] as well as other malignancies [14], yet the precise cfDNA metrics that are most clinically relevant remain controversial, possibly because of the heterogeneity of the study backgrounds, which vary regarding clinical stage, tumor pathology, blood sample origin and the method of cfDNA measurements. In the present study, we analyzed the cfDNA profile of RCC patients to determine whether cfDNA can be a promising tool for diagnosis as well as a prognostic factor for RCC. We demonstrated that the level of cfDNA was associated with high sensitivity and specificity for diagnosis of RCC. We also demonstrated that shorter fragment sizes of cfDNA were highly correlated with worse prognosis for RCC patients. Collectively, these markers may lead to better alternative tools to track the clinical course of RCC patients.

## RESULTS

### Patient characteristics

The clinicopathological characteristics are summarized in Table 1. In total, 92 RCC patients were histologically diagnosed as clear cell RCC and subsequently enrolled in this study. The RCC cohort consisted of 72 male and 20 female, and the median age was 68 years (range 23–90 years). Seventy-nine patients had no metastases, and 13 had metastases upon diagnosis of RCC (lung 10, lymph node 4, bone 2 and pancreas 1). The median follow-up duration was 6.8 months (range 0.5–22.1 months). Global median plasma cfDNA concentration of RCC patients and healthy controls were 17.0 ng/ml (range 4.8–69.6 ng/ml) and 19.2 ng/ml (range 4.5–45.2 ng/ml), respectively (*p =* 0.252), indicating no significant difference between these groups.

**Table 1:** Characteristics of RCC patients and healthy controls (*n* = 133)

Characteristics	Number of patients (%)				*p*-value
	RCC patients (*n* = 92)		Healthy controls (*n* = 41)		
Age: median (range) (years)	68	(23–90)	57	(26–79)	<0.001
Gender: Male/Female	72/20		24/17		0.021
WBC: median (range) (cells/mm3)	5640	(3050–12990)	5200	(3000–11780)	0.069
Hb: median (range) (g/dl)	13.7	(6.9–17.2)	13.9	(11.9–16.2)	0.481
Na: median (range) (mEq/l)	140	(130–146)	141	(139–144)	0.002
Alb: median (range) (g/dl)	4.0	(1.8–4.8)	4.1	(3.6–4.7)	0.095
CRP: median (range) (mg/dl)	0.06	(0–20.57)	0	(0–0.19)	<0.001
NLR: median (range)	2.61	(0.76–9.68)	2.16	(0.81–4.72)	0.012
Clinical stage					
I	58	(63.0%)			
II	4	(4.3%)			
III	15	(16.3%)			
IV	15	(16.3%)			
Clinical T stage					
1a	52	(56.5%)			
1b	10	(10.9%)			
2	6	(6.5%)			
≥3	24	(26.1%)			
Metastasis					
Negative	79	(85.9%)			
Positive	13	(14.1%)			
Fuhrman nuclear grade					
Low grade (without G3 and G4)	51	(55.4%)			
High grade (with G3 and G4)	17	(18.5%)			
Unknown	24	(26.1%)			
LVI					
Negative	50	(54.3%)			
Positive	18	(19.6%)			
Unknown	24	(26.1%)			
Follow-up term: median (range) (months)	6.8	(0.5–22.1)			
Disease progression	9	(9.8%)			
Cancer death	5	(5.4%)			

Abbreviations: WBC, white blood cell; Hb, hemoglobin; Alb, albumin; CRP, C-reactive protein; NLR, neutrophil to lymphocyte ratio; LVI, lymphovascular invasion.

### The levels of plasma cfDNA increased in RCC patients

**Figure 1:**
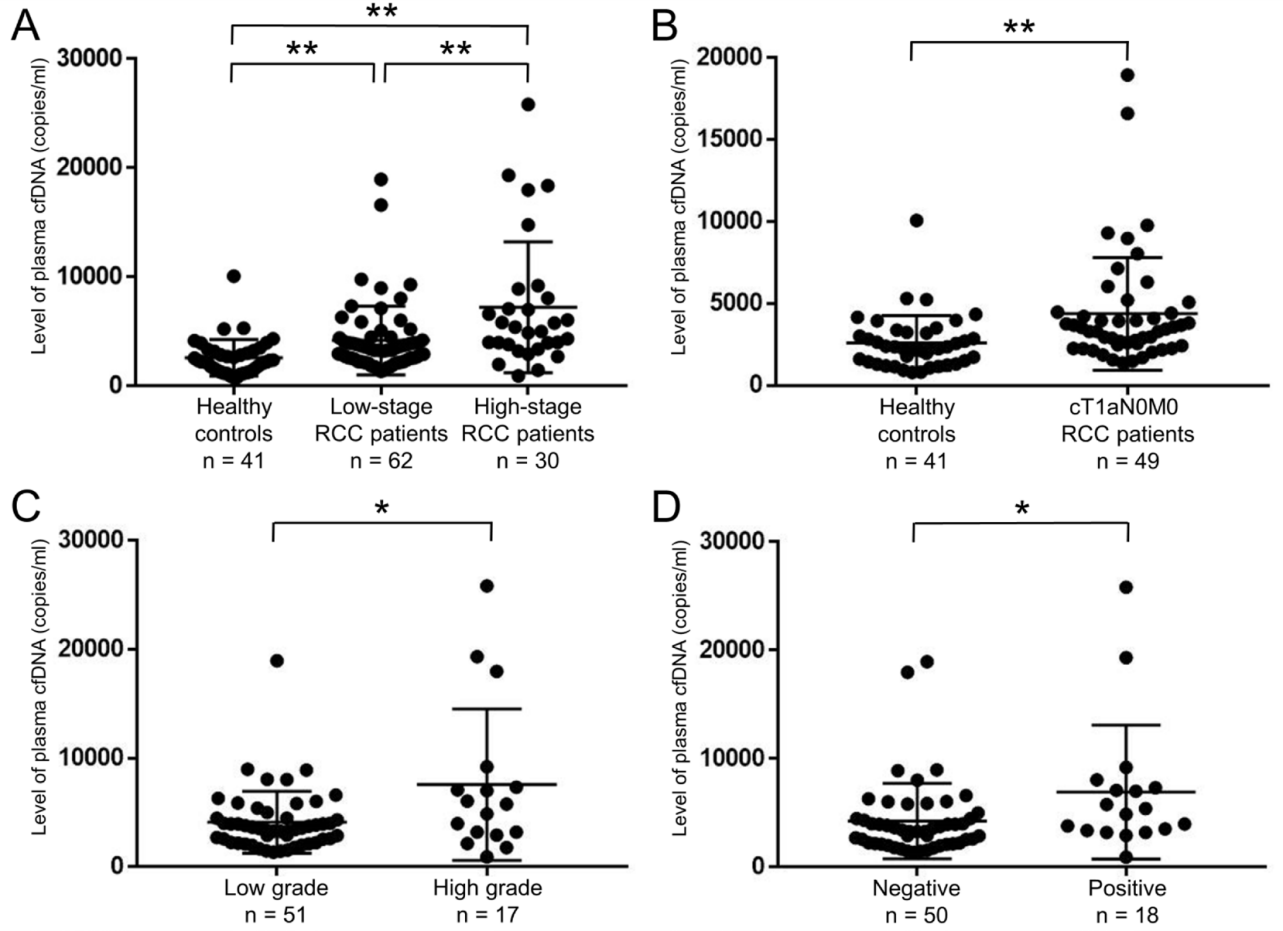
Level of plasma cfDNA was able to distinguish between healthy controls and RCC patients (**A**) Levels of plasma cfDNA were quantified by real-time PCR. Comparison of cfDNA levels among healthy controls (*n =* 41), low-stage (stage I–II) RCC patients (*n =* 62), and high-stage (stage III–IV) RCC patients (*n =* 30). ^**^p < 0.01 (Dunn’s multiple comparison test). (**B**) Comparison of cfDNA levels between healthy controls (*n =* 41) and cT1aN0M0 RCC patients (*n =* 49). ^**^p < 0.01 (Wilcoxon test). (**C**) Levels of plasma cfDNA were significantly higher with Fuhrman nuclear grade 3 and 4 (high grade) than without grade 3 and 4 (low grade) (*n =* 68). ^*^p < 0.05 (Wilcoxon test). (**D**) Levels of plasma cfDNA were significantly higher with positive LVI than negative (*n =* 68). ^*^p < 0.05 (Wilcoxon test).

To examine the levels in RCC patients, we performed real-time PCR on plasma cfDNA samples. Overall, the levels of plasma cfDNA from RCC patients (median 3803, range 936–25831 copies/ml) were significantly higher than those from healthy controls (median 2242, range 792–10081 copies/ml) (*p <* 0.001). Moreover, the levels of plasma cfDNA increased according to TNM stage (Figure 1A). Importantly, the elevated levels were confirmed even in plasma cfDNA of cT1aN0M0 RCC patients (median 3442, range 1368–18960 copies/ml) compared to healthy controls (median 2242, range 792–10081 copies/ml) (*p <* 0.001, Figure 1B). Regarding pathological status, the levels of plasma cfDNA from RCC patients with Fuhrman nuclear grade 3 and 4 (median 5796, range 936–25831 copies/ml) were higher than those without grade 3 and 4 (median 3547, range 1368–18960 copies/ml) (*p =* 0.048, Figure 1C). The levels of plasma cfDNA from RCC patients with lymphovascular invasion (LVI) (median 5159, range 936–25831 copies/ml) were also higher than those without LVI (median 3366, range 1368–18960 copies/ml) (*p =* 0.016, Figure 1D).

### The fragment size of plasma cfDNA was altered depending on the clinical characteristics of RCC patients

**Figure 2:**
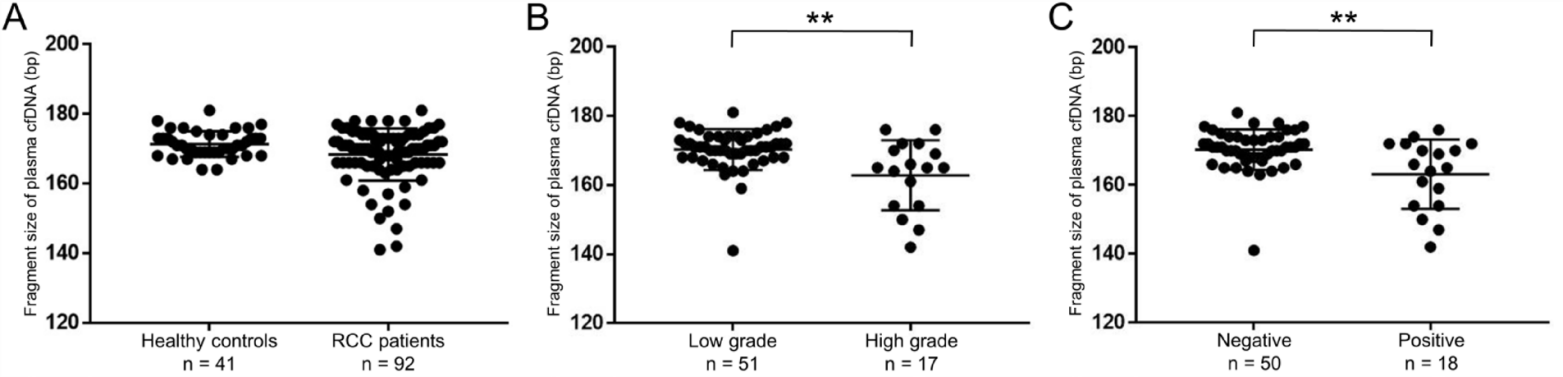
Fragment sizes of plasma cfDNA in RCC patients were shorter than those in healthy controls (**A**) The cfDNA fragment size was quantified by a microfluidics-based platform. Comparison of fragment sizes of plasma cfDNA between healthy controls (*n =* 41) and RCC patients (*n =* 92). (Wilcoxon test). (**B**) The cfDNA fragment size was significantly shorter with Fuhrman nuclear grade 3 and 4 (high grade) than without grade 3 and 4 (low grade) (*n =* 68). ^**^p < 0.01 (Wilcoxon test). (**C**) The cfDNA fragment size was significantly shorter with positive LVI than negative (*n =* 68). ^**^p < 0.01 (Wilcoxon test).

We further examined the fragment size of plasma cfDNA using a microfluidics-based platform. The fragment sizes of plasma cfDNA from RCC patients (median 170, range 141–181 bp) were shorter than those from healthy controls (median 171, range 164–181 bp), although there was no statistical difference between the two groups (*p =* 0.052, Figure 2A). Interestingly, considering the pathological status of RCC, the fragment sizes of plasma cfDNA from RCC patients with high Fuhrman nuclear grade or positive LVI were significantly shorter than those without both factors (both, *p <* 0.01, Figure 2B, 2C).

### ROC curve analysis to assess potential diagnostic marker of cfDNA level

**Figure 3:**
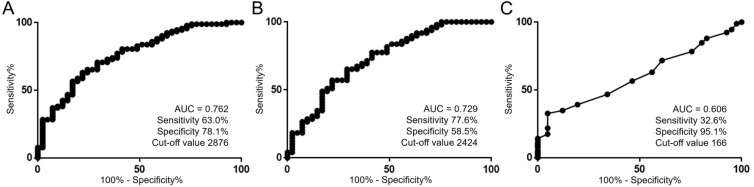
Utility of the level of plasma cfDNA as a diagnostic tool for RCC (**A**) ROC curve analysis for the diagnosis of RCC using cfDNA level (*n =* 133). The units for the cut-off value is copies/ml. (**B**) ROC curve analysis for the diagnosis of cT1aN0M0 RCC using cfDNA level (*n =* 90). The units for the cut-off value is copies/ml. (**C**) ROC curve analysis for the diagnosis of RCC using the cfDNA fragment size (*n =* 133). The units for the cut-off value is bp.

To further investigate the diagnostic capability of plasma cfDNA, we performed receiver operating characteristic (ROC) curve analysis on both the level and fragment size of cfDNA. ROC curve analysis revealed that the cfDNA level showed a sensitivity of 63.0% and a specificity of 78.1% to diagnose RCC (area under the curve (AUC) = 0.762, cut-off value 2876 copies/ml, *p <* 0.001, Figure 3A). Moreover, this metric even demonstrated a sensitivity of 77.6% and a specificity of 58.5% to diagnose the presence of cT1aN0M0 RCC (AUC = 0.729, cut-off value 2424 copies/ml, *p <* 0.001, Figure 3B). In contrast, ROC curve analysis using the cfDNA fragment size demonstrated a sensitivity of 32.6% and a specificity of 95.1% to diagnose the presence of RCC (AUC = 0.606, cut-off value 166 bp, *p =* 0.009, Figure 3C). Multivariate logistic regression analysis revealed that higher levels of plasma cfDNA were significantly associated with the diagnosis of RCC (odds ratios 1.734, 95% confidence interval 1.280–2.507, *p <* 0.001, Table 2). These results suggest that the level of plasma cfDNA is a potential biomarker for the diagnosis of RCC.

**Table 2:** Univariate and multivariate logistic regression analysis for the diagnosis of RCC (*n* = 133)

	Univariate			Multivariate		
	OR	95% CI	*p*-value	OR	95% CI	*p*-value
Age (years)	1.073	1.042–1.108	<0.001	1.080	1.045–1.122	<0.001
Gender	2.550	1.150–5.678	0.021	3.404	1.292–9.441	0.013
WBC (X 1000)	1.248	0.962–1.695	0.100			
Hb	0.857	0.671–1.063	0.167			
Na	0.757	0.612–0.913	0.003	-	-	-
Alb	0.338	0.098–0.912	0.030	-	-	-
CRP (X 0.1)	3.616	1.656–10.676	<0.001	-	-	-
NLR	1.757	1.160–2.936	0.005	-	-	-
Level of plasma cfDNA (X 1000)	1.694	1.298–2.344	<0.001	1.734	1.280–2.507	<0.001
CfDNA fragment size	0.914	0.841–0.980	0.009	-	-	-
Global cfDNA concentration	1.000	0.968–1.037	0.988			

Abbreviations: OR, odds ratio; CI, confidence interval; WBC, white blood cell; Hb, hemoglobin; Alb, albumin; CRP, C-reactive protein; NLR, neutrophil to lymphocyte ratio.

### The fragment size of plasma cfDNA was prognostic marker in RCC patients

**Figure 4:**
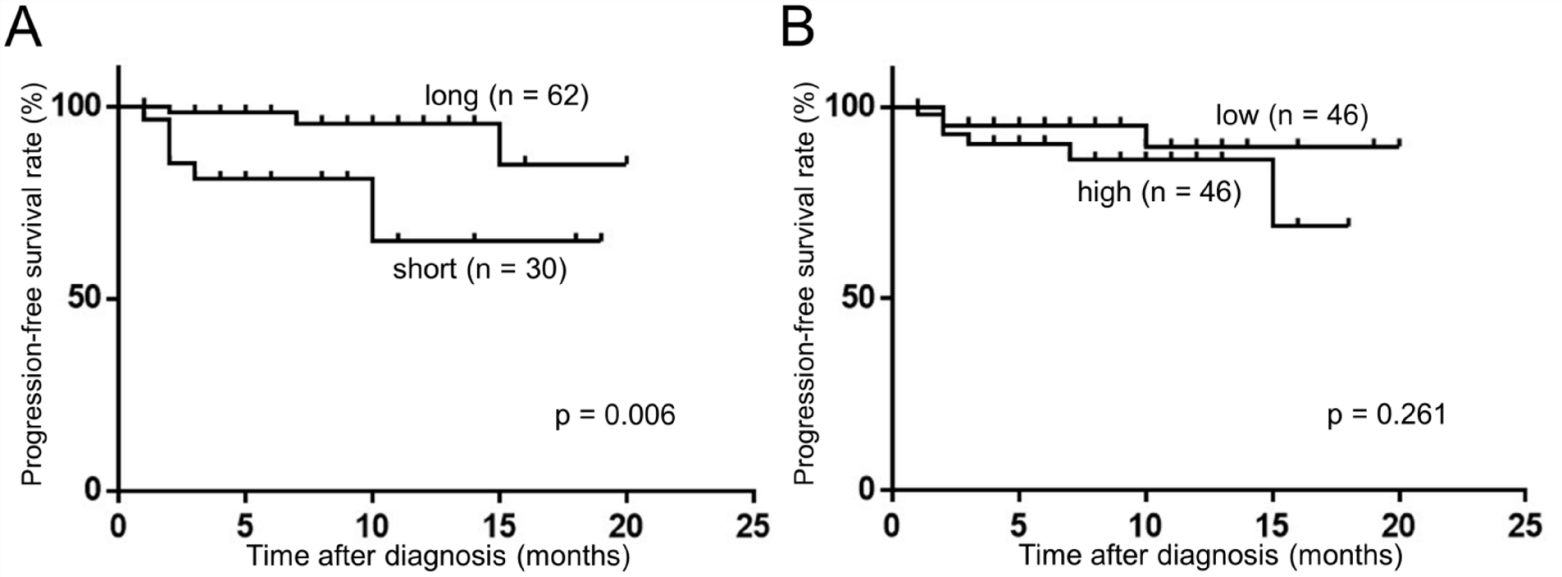
Fragment size of plasma cfDNA was associated with PFS. (Kaplan–Meier method and log-rank test) (**A**) The association of cfDNA fragment size between ≤166 bp (short) and >166 (long). (**B**) The association of cfDNA levels between ≤3803 copies/ml (median value of RCC patients) (low) and >3803 (high).

**Figure 5:**
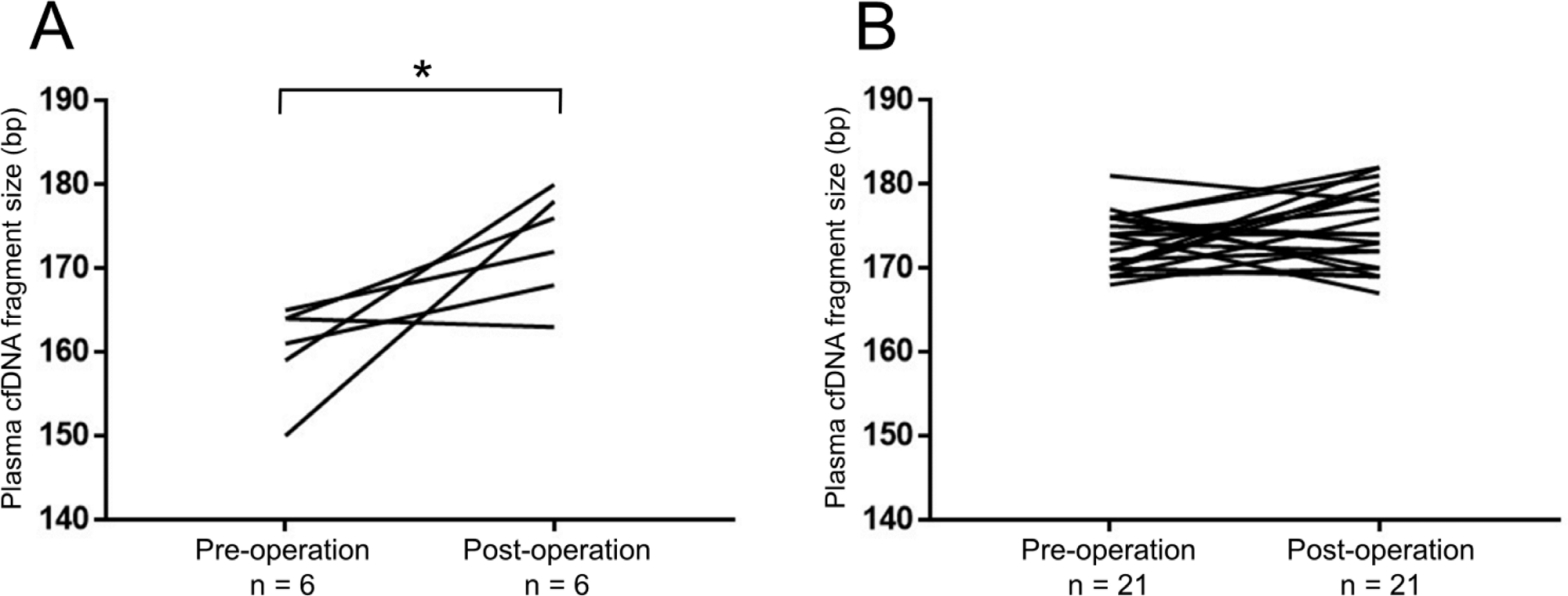
The cfDNA fragment size significantly increased after removal of the primary tumors in RCC patients with short fragment size (≤166 bp) Changes in the cfDNA fragment size before (pre-operation) and after (post-operation) surgical removal of the primary tumor were quantified by a microfluidics-based platform. ^*^p < 0.05 (Wilcoxon signed-rank test). (**A**) In RCC patients with short cfDNA fragment size (≤166 bp) at pre-operation (*n =* 6). (**B**) In RCC patients with long cfDNA fragment size (>166 bp) at pre-operation (*n =* 21).

We next evaluated whether the plasma cfDNA was correlated with prognosis in RCC patients. Using the Kaplan–Meyer method and log-rank test, we found that the cfDNA fragment size was significantly associated with progression-free survival (PFS) rate (long vs. short, *p =* 0.006, Figure 4A), although the levels of plasma cfDNA showed no significant association with PFS (low vs. high, *p =* 0.261, Figure 4B). Since the most prominent peak in the cfDNA fragment size profile was observed at 166 bp using next-generation sequencing methods [15–17], we divided our cohort into two groups, one with longer (> 166 bp) and one with shorter (≤ 166 bp) fragment sizes. We evaluated the cfDNA fragment size from RCC patients (*n =* 27, stage I: 11, stage II: 1, stage III: 6, stage IV: 9) before and after surgical removal of the primary tumor. Interestingly, the cfDNA fragment size was significantly longer after surgical removal in the “short” (≤ 166 bp) group (Figure 5A), whereas the cfDNA fragment size did not change in the “long” group (> 166 bp) (Figure 5B). These results indicate that the fragment size of plasma cfDNA was a prognostic marker of RCC.

## DISCUSSION

Currently, no reliable biomarkers for RCC have been identified that are both minimally invasive and informative for diagnosing early-stage disease. Recently, blood-based tests, also known as liquid biopsy, serve as potential alternatives to radiological tests and tissue biopsies. In particular, cfDNA is thought to reflect disease status [18] and has been shown to be advantageous for the diagnosis, prognosis and monitoring of several cancers [6]. For instance, cfDNA level was more accurate than classical tumor markers, such as carcinoembryonic antigen or carbohydrate antigen 19-9, in diagnosing colon cancer [19]. In addition to overall levels of cfDNA, another cfDNA parameter that may be clinically relevant is fragment size. Patients with melanoma and lung cancer had shorter fragment sizes of cfDNA than health controls [20], although this metric has been scarcely explored for clinical use. Accordingly, in this study we examined both the level and fragment sizes of cfDNA as potential novel markers for RCC.

Through the cfDNA analyses in this study, we have demonstrated several novel findings that may have utility in clinical settings. First, the level of plasma cfDNA can be applied as a diagnostic marker for RCC. The level of plasma cfDNA yielded a moderate AUC value of 0.762 for the diagnosis of RCC, much in line with previous reports [7, 9, 10]. Interestingly, the level yielded a mild AUC value (0.729) even for the diagnosis of cT1aN0M0 RCC. These results offer that cfDNA level is a helpful tool for the detection of RCC. Importantly, our data support the diagnostic potential of cfDNA level when assessed alongside other clinical parameters by multivariate analysis. Recent technological developments, such as digital PCR [21], could further facilitate determination of cfDNA level as absolute copy number. Future studies are needed to validate our results to diagnose RCC patients at earlier stages using such new technologies. Secondly, regarding the prognostic potential of plasma cfDNA, RCC patients in the “short cfDNA fragments” group (≤166 bp) showed a significant association with worse PFS, and this is consistent with our findings that shorter cfDNA fragment size was associated with more aggressive pathological features. In hepatocellular carcinoma patients, the size distribution of plasma cfDNA shifted to shorter fragments with an increasing proportion of tumor-derived DNA [15]. Combined with the previous report [15], our results suggested that the “short cfDNA fragments” group could have a greater proportion of tumor-derived DNA, indicative of greater tumor burden, and thus correlating with worse PFS. Of course, further studies are needed to examine this phenomenon.

There are some apparent limitations in this study. Our study was retrospective and had relatively short follow-up duration. Further investigations are needed to validate our results in larger numbers of patient by multi-institutional studies.

In conclusion, our results imply that the level and fragment size of plasma cfDNA have promising diagnostic and prognostic potential in RCC patients, respectively. Given that plasma cfDNA is easily collected from peripheral blood, these newly discovered markers can be convenient and precise tools for understanding RCC.

## MATERIALS AND METHODS

### Study design

Between June 2015 and June 2017, a total of 92 patients with clear cell RCC were enrolled in this study. None of the patients had received systemic therapy such as molecular-targeted therapies or immunotherapies. All patients had no sign of other active cancers in study period. This study was approved by the Institutional Review Board of Osaka University Hospital (3397-12). All patients had provided written informed consent for the collection and analysis of blood samples.

Clinically, PFS was evaluated from the first blood sampling day to the last follow-up point or the detection of a progressive event according to RECIST 1.1 criteria on a computer tomography scan [22] on all RCC patients, irrespective of clinical metastasis status or whether surgical removal was performed. All patients were pathologically diagnosed by surgical resection sample or needle biopsy. Histological diagnosis was determined on the basis of standard hematoxylin- and eosin-stained sections. Two or more experienced senior pathologists assessed the pathological diagnosis by 7th American Joint Committee on Cancer TNM staging system [23], Fuhrman nuclear grade [24] and LVI [25]. Fuhrman nuclear grade and LVI were only assessed in surgical removal specimens.

### Preparation of blood samples and cfDNA extraction from plasma

Whole blood (2.0–7.0 ml) was collected directly into EDTA tubes. Within three hours after collection, all blood samples were centrifuged sequentially at 900 and 20,000 gravity for 10 minutes each, and supernatants were stored at –80° C as plasma. Post-operative blood samples (*n =* 27) were collected at least 1 month after surgical resection of primary tumors to avoid the effect of surgical stress on cfDNA characteristics. CfDNA was isolated from 0.8–3.0 ml plasma samples using the QIAamp^®^ Circulating Nucleic Acid Kit (QIAGEN, Hilden, Germany) according to the manufacturer’s protocol.

### Quantification of cfDNA levels

Quantitative real-time PCR analysis was performed using a CFX Connect^™^ Real-Time System (Bio-Rad Laboratories, Hercules, CA, USA) to detect levels of plasma cfDNA. ACTB was used as the target for quantification of cfDNA fragments with an amplicon length of 106 bp [26]. Plasmid DNA that included the ACTB target locus was prepared using a TA-cloning method with the TA-Enhancer Cloning Kit (NIPPON GENE, Tokyo, Japan) and this plasmid construct was then used as a traceable standard for all measurements. 1,000,000 copies of plasmid standards were converted into absolute copy number as calculated from molecular weight. Final reaction volumes of 10 μl were used, consisting of 5 μl of SsoAdvanced^™^ Universal SYBR^®^ Green Supermix (Bio-Rad Laboratories, Hercules, CA, USA) and 350 pmol/ml forward and reverse primers (forward: 5′-TCGTGCGTGACATTAAGGAG; reverse: 5′-GGCAGCTCGTAGCTCTTCTC) [26]. Standard curves were calculated using serial dilutions of plasmid DNA, allowing determination of cfDNA levels from 1 ml of plasma.

### Measurement of global concentration and fragment size of cfDNA

Global cfDNA concentration from 1 ml plasma was measured using the Qubit^®^ 2.0 Fluorometer (Thermo Fisher Scientific, Waltham, MA, USA). The cfDNA fragment size was measured using a microfluidics-based platform, the Agilent 2100 Bioanalyzer with the High Sensitivity DNA Kit (Agilent Technologies, Santa Clara, CA, USA). The Agilent 2100 Expert software (version B.02.08) offers a smear analysis with an integrator feature that allows precise measurement of the smear region. The software automatically determines the mean size for each defined smear region of plasma cfDNA.

### Statistical analysis

Statistical analysis was performed using JMP^®^ Pro 12.2.0 (SAS Institute Inc., Cary, NC, USA). Results on patient and cfDNA characteristics are presented as median + range, and data was compared using *χ*^2^-test, Wilcoxon test, Wilcoxon signed-rank test or Dunn’s multiple comparison test. ROC curve analysis was used to generate AUC values to evaluate cfDNA characteristics’ predictive ability for the diagnosis of RCC. Univariate and multivariate logistic regression analysis were performed to assess the relative contributions of various factors (age, gender, white blood cell count, hemoglobin, sodium, albumin, C-reactive protein and neutrophil to lymphocyte ratio) and plasma cfDNA characteristics such as level, fragment size and global concentration for the diagnosis of RCC. PFS rate was calculated using the Kaplan–Meier method. Differences among the two groups were assessed by log-rank test and were considered statistically significant when the *p*-value was less than 0.05.

## Abbreviations

AUC: area under the curve
cfDNA: cell-free DNA
LVI: lymphovascular invasion
PFS: progression-free survival
RCC: renal cell carcinoma
ROC: receiver operating characteristic
